# 

**Published:** 1892-10

**Authors:** 


**Special Notice.**—The American Public Health Association will meet in the City of Mexico on the 29th of November, and hold four days. Excursion rate tickets good for thirty days will be on sale at all offices. A special train for physicians will pass through Austin on the morning of the 22nd, stopping over one day at San Antonio. If a sufficient number of physicians wish to go and will make up their party, a special Pullman car can be secured for the through trip by writing to Maj. Lewis, the agent, at Driskill hotel, Austin.

